# Perioperative Outcome of Robotic Approach *Versus* Manual Videothoracoscopic Major Resection in Patients Affected by Early Lung Cancer: Results of a Randomized Multicentric Study (ROMAN Study)

**DOI:** 10.3389/fonc.2021.726408

**Published:** 2021-09-09

**Authors:** Giulia Veronesi, Abbas El-Sayed Abbas, Piergiorgio Muriana, Rosalba Lembo, Edoardo Bottoni, Gianluca Perroni, Alberto Testori, Elisa Dieci, Charles T. Bakhos, Shamus Car, Luca Luzzi, Marco Alloisio, Pierluigi Novellis

**Affiliations:** ^1^Department of Thoracic Surgery, IRCCS San Raffaele Scientific Institute, Milan, Italy; ^2^Faculty of Medicine and Surgery, Vita-Salute San Raffaele University, Milan, Italy; ^3^Department of Thoracic Medicine and Surgery, Lewis Katz School of Medicine, Temple University Hospital, Philadelphia, PA, United States; ^4^Department of Surgery, Lewis Katz School of Medicine, Temple University Hospital, Philadelphia, PA, United States; ^5^Department of Anesthesia and Intensive Care, IRCCS San Raffaele Scientific Institute, Milan, Italy; ^6^Division of Thoracic and General Surgery, Humanitas Clinical and Research Center, Rozzano, Italy; ^7^Division of Thoracic Surgery, Department of Surgery, University of Maryland School of Medicine, Baltimore, MD, United States; ^8^Thoracic Surgery Unit, Department of Medicine, Surgery and Neuro Sciences, Diagnostic Imaging, University of Siena, Azienda Ospedaliera Universitaria Senese, Siena, Italy; ^9^Department of Biomedical Science, Humanitas University, Rozzano, Italy

**Keywords:** non-small cell lung cancer (NSCLC), surgery, robotic surgery, VATS, randomized study

## Abstract

**Introduction:**

We report the results of the first prospective international randomized control trial to compare the perioperative outcome and surgical radicality of the robotic approach with those of traditional video-assisted surgery in the treatment of early-stage lung cancer.

**Methods:**

Patients with clinical stage T1–T2, N0–N1 non-small cell lung cancer (NSCLC) were randomly assigned to robotic-assisted thoracoscopic surgery (RATS) or video-assisted thoracic surgery (VATS) resection arms. The primary objective was the incidence of adverse events including complications and conversion to thoracotomy. The secondary objectives included extent of lymph node (LN) dissection and other indicators.

**Results:**

This trial was closed at 83 cases as the probability of concluding in favor of the robot arm for the primary outcome was null according to the observed trend. In this study, we report the results of the analysis conducted on the patients enrolled until trial suspension. Thirty-nine cases were randomized in the VATS arm and 38 in the robotic arm. Six patients were excluded from analysis. Despite finding no difference between the two arms in perioperative complications, conversions, duration of surgery, or duration of postoperative stay, a significantly greater degree of LN assessment by the robotic technique was observed in regards to the median number of sampled LN stations [6, interquartile range (IQR) 4–6 *vs*. 4, IQR 3–5; *p* = 0.0002], hilar LNs (7, IQR 5–10 *vs*. 4, IQR 2–7; *p* = 0.0003), and mediastinal LNs (7, IQR 5–10 *vs*. 5, IQR 3–7; *p* = 0.0001).

**Conclusions:**

The results of this trial demonstrated that RATS was not superior to VATS considering the perioperative outcome for early-stage NSCLC, but the robotic approach allowed an improvement of LN dissection. Further studies are suggested to validate the results of this trial.

**Clinical Trial Registration:**

clinicaltrials.gov, identifier NCT02804893.

## Introduction

The robotic-assisted thoracoscopic surgery (RATS) approach has emerged as a valid alternative to the traditional minimally invasive video-assisted thoracoscopic surgery (VATS) (1–3). Thanks to significant technical advantages and stereoscopic visualization, it has become the preferred technique of an increasing number of thoracic surgeons (4). Many studies have shown that robotic-assisted pulmonary resection is both feasible and safe for the treatment of lung cancer (1–3, 5–7), with long-term outcomes comparable to that reported for open and VATS approaches (8, 9).

Some retrospective analyses of population-based database showed that RATS was associated with improved perioperative outcomes compared to the open approach but had comparable results to VATS (10, 11). In 2016, Agzarian et al. conducted a comparative meta-analysis of robotic pulmonary resection and other modalities. There were no significant differences in conversion rates, prolonged air leaks, blood loss, or length of stay between RATS and VATS (12). Different results were shown in 2017 by Oh et al., who analyzed the Premier Healthcare Database to compare perioperative clinical outcomes from elective lobectomy by RATS, VATS, and thoracotomy, with propensity score matching (1:1). Compared with the VATS and open approaches, RATS lobectomy was associated with a shorter length of stay, lower complication rates, and lower conversion rate (10).

Recently, Kneuertz et al. showed that lymph node upstaging with RATS was superior to VATS and comparable to the open approach (13). Novellis *et al.* also reported a retrospective comparative analysis of RATS *versus* open and VATS approaches for lung lobectomies, with a significant difference in perioperative outcome in favor of the robotic approach (11). Their study also observed that more lymph node (LN) stations were removed by RATS when compared with VATS and thoracotomy (11).

To date, no randomized trials comparing the early- and long-term outcome of VATS *versus* RATS lobectomy have been reported. We therefore designed a multicenter randomized controlled trial with the primary objective to assess the overall perioperative complication rate, including conversion to thoracotomy and 30-day complication rate. As a secondary objective, we explored the extent of LN dissection, postoperative hospital stay, duration of surgery, long-term assessment of pain, quality of life (QoL), and recurrence rate. In this paper, we report the results of the early outcomes.

## Materials and Methods

### Ethics Committee Approval

The study protocol was evaluated by the Humanitas Clinical and Research Center Ethic Committee (no. 1566) and approved by the local internal review boards of all participating centers. It was registered at ClinicalTrials.gov (NCT02804893). All participants gave written informed consent to participate in the study.

### Study Design

We designed a prospective, randomized, multicenter study on 300 patients (150 VATS lobectomies and 150 RATS lobectomies) affected by early-stage non-small cell lung cancer (NSCLC). The expected time period for recruitment was 1 year, and that for follow-up was 2 years. For participation in the study, trial surgeons needed a minimum of 30 major lung resections performed using one or each of the two techniques. Every participating center needed the ability to offer both techniques (RATS and VATS).

Randomization was performed through a dedicated Internet-based system with a balance software for center stratification (validated by FDA, Title 21 of the Code of Federal Regulations, Part 11) within 4 weeks prior to the planned operation date once the eligibility of the patient had been confirmed and consent was given. This interval allowed a sufficient time to schedule the date of surgery.

### Study Objectives

The aim of this study was to compare VATS and RATS approaches in the treatment of early-stage NSCLC in terms of operative and perioperative results. We identified as primary endpoints the rate of conversions, bleeding, and perioperative complications (assessed by modified Clavien–Dindo scale). The secondary endpoints were duration of surgery, number of resected LNs, number of dissected LN stations, postoperative hospital stay, postoperative pain with daily evaluation, quality of life by EORTC QoL-C30, postoperative respiratory function, and rate of local or distant recurrence at 2 years.

### Inclusion/Exclusion Criteria

The inclusion criteria were as follows: age older than 18 years old and known or suspected NSCLC. In case of suspected lung cancer with no preoperative diagnosis, frozen section was indicated during surgery in order to confirm the disease. If a benign lesion was diagnosed, the patient was considered a dropout of the study. Other inclusion criteria include the following: patients in clinical stage T1–T2–T3, N0–N1, candidate for lobectomy, anatomical segmentectomy, or bilobectomy; patients with multiple lung tumors could be included if they could be resected with a lobectomy, lobectomy plus segmentectomy, or bilobectomy and each tumor should be staged separately; and American Society of Anesthesiologists score 1–3. Written informed consent was signed prior to performing any study procedures.

The exclusion criteria were also as follows: metastatic cancer, extrapulmonary primary cancers in the past 2 years, severe heart disease, alcohol abuse, renal impairment (creatinine >2.5 mg/dl), and other serious comorbidities that contraindicate surgery.

### Preoperative Evaluation

Preoperative analysis included staging studies such as chest CT scan and PET scan. For stages higher than IA, brain CT with contrast or MRI was required, while brain MRI was done in case of suspicious brain lesions. Standard functional evaluation included EKG, cardiological evaluation, pulmonary function tests, and anesthesia evaluation. When required by the physician, additional tests were introduced, such as cardiac stress test, echocardiography, and pulmonary scintigraphy.

Staging and functional exams were done within 6 weeks of surgery. In case of suspicious mediastinal nodes, endobronchial ultrasound or mediastinoscopy was done before resection.

During the operation, frozen section for confirmation of diagnosis was done in cases of lesions with no preoperative diagnosis. All operations were performed under general anesthesia, with the patients in the lateral decubitus position.

### Operative Approaches

VATS lobectomy or segmentectomy was performed through one to four thoracoscopic incisions without rib spreading. The procedure was performed with videoscopic visualization without direct vision. The hilar structures were dissected, stapled, and divided. Endoscopic ligation of pulmonary arterial branches was occasionally performed. The fissure was completed, and the lobe of lung was resected. This definition of VATS lobectomy is a modification of CALGB 39802 (14).

Robotic lobectomy or segmentectomy was performed through four to five thoracoscopic incisions without rib spreading. The Da Vinci Robotic System (Intuitive, Sunnyvale, USA) was used. Under 3D vision, the hilar structures (vein, artery, and bronchus) were dissected, ligated, and divided in sequence using ligatures, by oversewing, or with staplers. The surgical approach for robotic resection was chosen according to the preference of the operator. In complete portal robotic lobectomy, all the ports were placed along a single intercostal space, and dissection was carried in a posterior to anterior direction with carbon dioxide use. The surgical specimen was then removed through a trans- or supradiaphragmatic incision (2). The robotic-assisted lobectomy approach was carried out through a utility incision at the fourth intercostal space and three additional ports without CO_2_ use. In this case, pulmonary hilum was approached from its anterior aspect. The specimen was extracted through the utility incision at the end of operation (1, 3).

LN dissection, both in VATS and RATS, was undertaken in accordance with the International Association of the Study of Lung Cancer recommendations of a minimum of six LN stations removed, of which three are from the mediastinum that includes the subcarinal station (15).

### Postoperative Care

The bladder catheter, if used, was removed when the urine output was adequate (>40 ml/h after surgery), without a known prostate disease. The chest tube was removed when the amount of drainage was less than 350 cc over 24 h (regardless of postoperative day) and in the absence of air leak. If prolonged air leak was observed, Heimlich valve was applied, and discharge was scheduled in the absence of clinical contraindications.

### Statistical Analysis

The primary objective was the incidence of adverse events including complications and conversions. At least one of these events was considered a failure of surgery. To have 80% power and a significance level of 5% to demonstrate a reduction of 15% rate of adverse events starting from 35% with VATS to 20% with robotic approach, a sample size of 300 subjects was initially calculated, 150 in each arm, with an expected dropout of less than 1% of the enrolled subjects. This sample size also had a power of 95% to detect a difference of 0.4 in the mean number of mediastinal lymph node stations, starting from 2.5, with a common standard deviation of 1.

Intention-to-treat and per-protocol analyses were performed. No imputation for missing data was planned. We also performed a planned *post-hoc* power analysis for secondary outcome, specifically for the number of hilar and mediastinal lymph nodes, and lymph node stations were harvested.

Categorical data were presented as absolute number and percentages and were compared by two-tailed *χ* ^2^ test or Fisher’s exact test when appropriate. Means and standard deviations were used when the variables were normally distributed, while medians and interquartile ranges were used with nonnormally distributed variables. Continuous measurements were compared using a nonparametric test or Student’s *t*-test if data were normally distributed. A logistic regression model with stepwise selection was used to identify predictors of primary outcome. Clinical data collected before randomization were entered into the model if they had a univariate *P*-value of less than 0.25. The trial group (robot *vs*. VATS) was forced into the multivariate model. Collinearity and overfitting were assessed with the use of a stepwise regression model and a Pearson correlation test. In the multivariate analyses, clinical factors or potential confounding variables were expressed as odds ratios with 95% confidence intervals. Statistical significance was set at the two-tailed 0.05 alpha level. All statistical analyses were performed with the Stata software (ver. 16; Texas USA).

## Results

**Figure 1:**
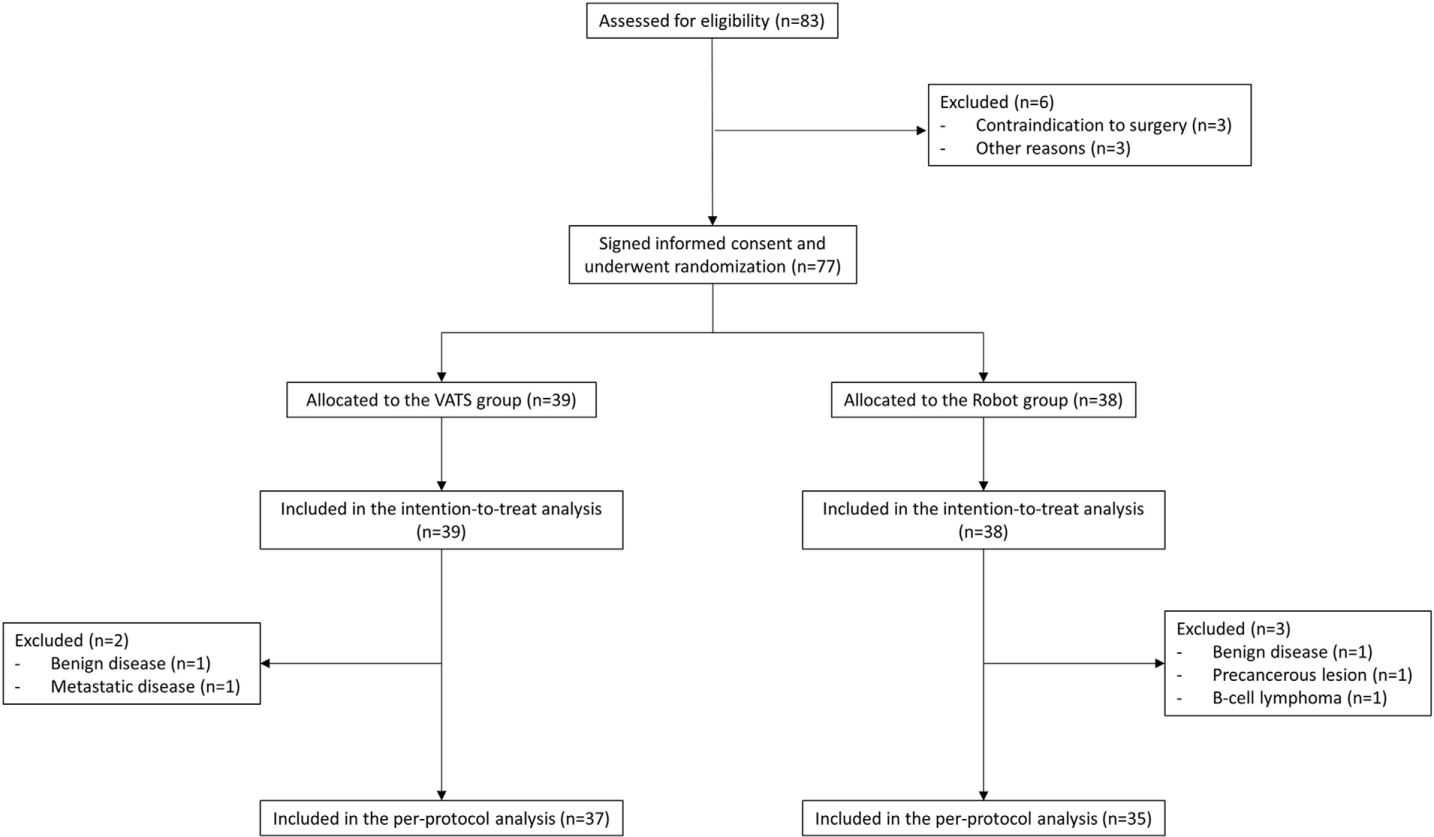
CONSORT flow diagram of enrollment, randomization, and analysis.

From April 2017 to November 2018, we screened 83 patients in four centers for eligibility (49 in center no. 1, 30 in center no. 2, and two patients both in center no. 3 and no. 4). Six patients were excluded from randomization: in detail, three patients did not undergo surgery because of contraindications encountered during the preoperative evaluation and three patients for other reasons. Seventy-seven patients provided informed consent and were randomized; 39 (51%) were assigned to the VATS group and 38 (49%) to the robot group (**Figure 1**). Patient demographics and disease characteristics were well balanced by treatment and are summarized in **Table 1**. The intraoperative results are reported in **Table 2**.

**Table 1:** Baseline characteristics of the patients enrolled in the VATS and ROBOT groups.

	Group VATS, *N* = 39	Group ROBOT, *N* = 38	*P*-value
Age, years (mean ± SD)	69 ± 7.3	69 ± 8.3	0.87
Female (%)	16 (41)	17 (45)	0.82
BMI (mean ± SD)	26 ± 4.1	27 ± 4.0	0.44
Smoking status			
Nonsmokers (%)	10 (38)	10 (45)	0.77
Former (%)	13 (45)	16 (57)	0.43
Stop smoking, years median (IQR)	15 (5–25)	20 (5–30)	0.90
Smokers (%)	16 (62)	12 (55)	0.77
Number of cigarettes/day median (IQR)	20 (20–30)	20 (10–30)	0.21
Pulmonary function evaluation			
FEV1, L (mean ± SD)	91 ± 24.8	86 ± 25.0	0.37
DLCO, mmol/min/KPa/L (mean ± SD)	76 ± 19.6	76 ± 20.5	0.91
ASA score (%)^a^			
I – II	24 (62)	19 (54)	0.64
III	15 (38)	16 (46)	
Clinical stage (%)^b^			
IA	25 (71)	28 (76)	0.48
IB	7 (20)	7 (19)	
IIA	1 (3)	2 (5)	
IIB	2 (6)	0 (0)	

SD, standard deviation; IQR, interquartile range; BMI, body mass index; FEV1, forced expiratory volume in the first second; DLCO, diffusing capacity of the lung for carbon monoxide; ASA score, American Society of Anesthesiology score.

a Data were available for analysis in 74 patients.

b Data were available for analysis in 72 patients.

**Table 2:** Intraoperative characteristics in the VATS and ROBOT groups of patients.

	Group VATS, *N* = 39	Group ROBOT, *N* = 38	*P*-value
Left side (%)	16 (41)	14 (37)	0.71
Lobe (%)			
Lower	16 (41)	16 (42)	0.92
Middle	1 (2.6)	6 (16)	0.056
Upper	22 (56)	16 (42)	0.21
Number of incisions, median (IQR)	2 (2–3)	4 (4–4)	<0.0001
Utility incision size, cm (mean ± SD)	3.3 ± 0.67	2.7 ± 0.86	0.01
Pleural adhesions (%)^a^			
Light	16 (76)	14 (64)	0.37
Moderate	5 (24)	3 (14)	0.39
Strong	0 (0)	5 (22)	0.02
Resection (%)			
Lobectomy	37 (95)	36 (95)	0.99
Segmentectomy	2 (5.1)	2 (5.3)	
R0 (%)^b^	38 (97)	38 (100)	0.15
R1 (%)	1 (3)	0 (0)	0.32
Operative time (skin to skin), min (mean ± SD)	183 ± 40.9	179 ± 54.2	0.71

SD, standard deviation; IQR, interquartile range.

a Data were available for analysis in 43 patients

b Radicality (R) was assessed following the definition proposed by the International Association of the Study of Lung Cancer (16, 17).

The study was closed as part of the periodic analyses by the independent data monitoring committee because, during the review, any difference observed between arms in terms of adverse events and the probability of concluding in favor of the robot arm was 0% (futility reason) if the observed trend had continued. In detail, conversion to thoracotomy was required in three cases of the RATS group and in two patients of the VATS group (*p* = 0.64). Early postoperative complications occurred in 13 cases (34%) in the robotic group and in nine cases (23%) in the VATS group (*p* = 0.28). Other post-procedural data are shown in **Table 3**.

**Table 3:** Postoperative outcomes and pathological results in the VATS and ROBOT groups of patients.

	Group VATS, *N* = 39	Group ROBOT, *N* = 38	*P*-value
Final pathology report (%)			
Adenocarcinoma	31 (79)	26 (70)	0.43
Squamous cell carcinoma	3 (8)	6 (16)	0.25
Other	5 (13)	5 (14)	0.66
Pathological stage (%)^a^			
IA	20 (58)	24 (69)	0.58
IB	7 (20)	4 (11)	
IIA	0 (0)	1 (3)	
IIB	4 (11)	4 (11)	
IIIA	4 (11)	2 (6)	
Size, mm median (IQR)	21 (14–30)	20 (15–28)	0.42
Number of hilar lymph nodes			
Mean ± SD	4.5 ± 3.6	7.8 ± 4.3	0.0006
Median (IQR)	4 (2–7)	7 (5–10)	0.0003
Number of mediastinal lymph nodes			
Mean ± SD	5.7 ± 3.7	8.1 ± 5.4	0.0001
Median (IQR)	5 (3–7)	7 (5–10)	0.0001
Number of lymph node stations sampled			
Mean ± SD	3.9 ± 1.2	5.2 ± 1.4	0.0001
Median (IQR)	4 (3–5)	6 (4–6)	0.0002
ICU recovery (%)	5 (13)	4 (11)	0.79
ICU stay, days median (IQR)	1 (1–1)	1 (1–1)	0.88
Chest tube duration, days median (IQR)	4 (3–6)	4 (3–6)	0.48
Hospital stay, days median (IQR)	4 (3–6)	5 (4–8)	0.27
Primary outcome^b^ (%)	11 (28)	16 (42)	0.24
Conversion to OPEN (%)	2 (5)	3 (8)	0.64
Early post-operative complications (%)	9 (23)	13 (34)	0.28
Complication grade (%)			
I–II	4 (12)	11 (32)	0.04
III	3 (9)	2 (8)	0.85
Most frequent early complication (%)			
Air leak	4 (10)	6 (16)	0.47
Atrial fibrillation	3 (7.7)	4 (11)	0.71
Serous drainage	1 (3)	1 (3)	0.99
Pneumonia	1 (3)	4 (11)	0.16
Pneumothorax	1 (3)	0 (0)	0.32
Atelectasis	1 (3)	3 (8)	0.29
Urinary tract infection	0 (0)	1 (3)	0.31
Other complications	2 (5)	3 (8)	0.62
Follow-up			
Adjuvant therapy^c^	4 (12)	3 (9)	0.69
Chemotherapy	4 (12)	3 (9)	0.69
Radiotherapy	2 (6)	2 (6)	0.99
Readmission (%)	0 (0)	4 (16)	0.08
Later complication (%)	2 (11)	5 (23)	0.33

SD, standard deviation; IQR, interquartile range; ICU, intensive care unit.

a Data were available for analysis in 70 patients.

b Composite outcome: conversion to open and/or any early postoperative complication.

c Data were available for analysis in 68 patients.

There was a substantial efficacy improvement in the robot arm for the secondary outcome especially for LN dissection parameters. The *post-hoc* analysis for this secondary outcome showed a power of 99% when comparing the mean number of LN station harvest and 94 and 60% for hilar and mediastinal LNs, respectively. A significant difference was found between the groups when the numbers of LNs and nodal stations harvested were considered. RATS was superior to VATS in terms of hilar (7, IQR 5–10 *vs*. 4, IQR 2–7; *p* = 0.0003) and mediastinal (7, IQR 5–10 *vs*. 5, IQR 3–7; *p* = 0.0001) LNs and in terms of nodal stations harvested (6, IQR 4–6 *vs*. 4, IQR 3–5; *p* = 0.0002).

Overall, the pathological examination showed a higher stage of disease than those predicted by preoperative evaluation in 15 patients; three additional patients were downstaged. Among patients that were upstaged, nine (25.7%) were enrolled in the VATS group and six (17.1%) in the robotic arm (*p* = 0.56). Nodal upstaging resulted evident in five patients (14.3%) treated by VATS (two from cN0 to pN1 and three from cN0 to pN2) and in four (11.4%) robotic cases (two from cN0 to pN1 and two from cN0 to pN2). No technique was found to be superior in terms of nodal upstaging (*p* = 0.72).

A univariate association between baseline variables on the primary outcome (perioperative complication including conversions) was performed; former smoker status, duration of smoking, and preoperative forced expiratory volume in the first second were statistically significant and were included in the multivariate analysis along with the randomization group. The logistic regression model showed that only the former smoker status was a statistically significant predictor of the primary outcome (OR 4.6; *p* = 0.03). However, this result was not further confirmed by a per-protocol analysis, probably due to the sample size (**Supplementary Table S1**).

Data on QoL, pain, and recurrence require a longer follow-up time to have a complete recording and are not reported in this initial analysis. The results of the per-protocol analysis are reported in **Supplementary Tables S1–S3**

## Discussion

In their systematic review of perioperative and oncological outcomes of patients undergoing surgical treatment of lung cancer, Azgarian and colleagues advocated the need of a prospective randomized trial to compare open surgery, VATS, and RATS to overcome biased results introduced by selection (12). On the other hand, Korst and Lee considered a randomized study between these approaches useless, as it would be a mere comparison of surgical instrumentation (18). Nevertheless, we believe that, in our present study, the risk of bias due to patient selection and preferences of the surgeon could be limited because all the enrolled individuals were treated in experienced centers offering both VATS and robotic surgery and after completion of the respective learning curves.

Two main results have been obtained by this prospective, multicentric, randomized trial: First, no statistical differences were found between RATS and VATS in terms of conversion rate and postoperative complications. Second, the robotic approach allowed an enhanced lymph node dissection compared to VATS.

Regarding the first objective of the study, the data are in line with previous retrospective nonrandomized trials (7). In a previous analysis by Novellis et al., a superiority of RATS *versus* VATS was reported mainly due to the different level of learning curve when the study was conducted (11). In this study, in order to avoid disparities in surgical experience, we defined a threshold of surgical procedures for each eligible thoracic surgeon with a minimum 30 cases of RATS and/or VATS based on learning curve thresholds previously described for those approaches (3, 19, 20). Despite the number of recruited subjects in the trial being lower than the expected target, the statistical analysis confirmed that the *post-hoc* power analysis based on the preliminary results was adequate to confirm similar outcome and safety of patients treated in the two arms.

This randomized study demonstrates that, for standard lobectomy, experienced surgeons can obtain similar results with both VATS and RATS approaches in terms of safety of the procedure. A prior meta-analysis of 12 retrospective studies showed no significant difference in conversion rate, pneumonia incidence, prolonged air leak, or arrhythmia between the two techniques (21). Swanson et al. performed a multihospital database analysis involving 15,502 patients: they compared wedge resection and lobectomy performed either by RATS or VATS after propensity score matching and found no differences in terms of complications up to 30 days between groups (22). Conversion from RATS to thoracotomy occurs, on average, in 6.7% of cases, with higher rates in left upper lobectomy (17.5%) and overall complication rate accounting for 42% (23, 24). As the probability of concluding in favor of the robot arm was 0% if the observed trend continued, we decided to close the study to new patient entry for “futility reasons”, upon the recommendation of the independent data monitoring committee.

Another result of this study relates to one of the secondary outcomes, observing a substantial improvement of efficacy in the RATS arm for the number of hilar LNs and LN stations harvested with a *post-hoc* power analysis of 94 and 99%, respectively. This finding is the first observation in a randomized trial of the superiority in number of hilar LNs and nodal stations (*p* = 0.0003 and *p* = 0.0002) harvested with the robotic approach compared with VATS. The mediastinal LN harvest was also significantly improved by the robotic technique (*p* = 0.0001), but a *post-hoc* analysis of 60% suggests that further investigation is needed.

In recent years, with the advent of minimally invasive surgery for lung cancer, the role of systematic mediastinal and hilar LN dissection has been investigated in depth. In fact, the presence of lymphatic involvement is one of the most impacting factors on the long-term survival of patients receiving surgery for NSCLC (25). In the Italian registry of VATS lobectomy, the number of resected LNs was noted as the only technical predictor of a nearly twofold probability of nodal upstaging in patients with clinical T1–T3, N0 NSCLC (26).

The term to describe the identification of unforeseen LN metastases at postoperative pathologic examination is nodal upstaging, which may be an indirect indicator of the oncological efficacy of the surgical technique. In our study cohort, both thoracoscopic and robotic techniques showed similar rates of nodal upstaging (14.3 *vs*. 11.4%, respectively), without a significant difference at statistical analysis (*p* = 0.72). Moreover, both approaches showed comparable ability to identify unanticipated hilar (N1) or mediastinal (N2) lymph node metastasis, yet there is no consensus on the performance of robotic surgery compared with VATS in terms of nodal upstaging.

In two propensity-matched analyses based on large samples including patients with clinical stage I tumors, contrasting results have been obtained (13, 27). In fact, in the study by Hennon et al. evaluating the impact of surgical approach on nodal upstaging in patients undergoing pulmonary lobectomy, the robotic technique was associated with slightly inferior results compared with VATS (11.2 *vs*. 11.7%, respectively) (27). On the other hand, in the study by Kneuertz and colleagues, robotic surgery had a significantly higher number of nodal upstaging than VATS (16.2 *vs*. 12.3%, *p* = 0.03) (13). According to our results, we cannot conclude about the superiority of one technique in terms of nodal upstaging due to the limited number of events. Future studies specifically designed to address this topic should be recommended in the future.

A large meta-analysis by Zhang et al. showed that VATS lymphadenectomy harvested a lower overall number of lymph nodes compared with patients treated by open thoracotomy, along with the resection of a lower number of N2 lymph nodes (28). According to the authors, such disparity may be caused by VATS surgeons wishing to avoid possible complications during mediastinal dissection.

Another previous retrospective series comparing LN dissection in VATS and RATS had controversial results. In a 2016 retrospective analysis, Toker et al. demonstrated a superiority of RATS in the number of N1 LNs harvested above station 11. However, no difference was found when N2 or station 10 was considered nor in the number of nodal stations dissected (29). Conversely, a recent meta-analysis involving 20 retrospective studies found no difference in the number of removed LNs (30).

In the present study, we found a median number of seven (IQR 5–10) hilar lymph nodes with RATS and four (IQR 2–7) with VATS (*p* = 0.0003) and seven (IQR 5–10) mediastinal lymph nodes *vs*. five (IQR 3–7) in VATS (*p* = 0.0001). Our data confirm previous retrospective studies, possibly related to the technical benefits of 3D vision and wristed instrumentation of the robotic platform over VATS (11, 15). Compared with VATS, the robotic system offers the possibility of better dissection of lymphatic structures despite the presence of fibrosis and enhanced control of hemostasis and lymphatic leakage (31). In the study by Merritt *et al.*, it was demonstrated that experienced surgeons are able to resect a higher number of overall and N2 lymph nodes by RATS compared with a group of patients treated by VATS (32). Nevertheless, the increased rate of lymph node dissection obtained in the robotic group was not associated to a higher incidence of complications, with particular regard to postoperative air leaks (*p* = 0.47) and serous chest drain (*p* = 0.99), despite a higher number of patients affected by strong pleural adhesions (22%) compared to cases treated by VATS (0%, *p* = 0.02). Moreover, complications occurring in the robotic group required no intervention in most cases (Clavien–Dindo grade I–II, *p* = 0.04). These results were consistent with a recent large meta-analysis by Ma et al. that showed better lymph node assessment, a reduction of 50% of the risk of conversion, and lower overall postoperative complication rate in patients undergoing pulmonary lobectomy by the robotic technique than VATS (33).

The technical advantages of robotic surgery have also been demonstrated for the treatment of locally advanced disease. In 2018, Veronesi et al. presented the results of a multicentric study of patients with stage IIIA disease who underwent robotic lung resection (34). Interestingly, in patients who had undergone preoperative induction therapy, the mean number of LNs harvested during the procedure as well as the rate of conversion to thoracotomy and postoperative complications did not differ from the upfront surgery group.

A growing number of studies have analyzed the oncological efficacy of parenchymal sparing resections in patients with early-stage NSCLC (35). The results of our study gain importance if they also translate to sublobar anatomical resections. It has been recently demonstrated that long-term survival in patients undergoing segmentectomy or lobectomy is still overlapping even in the presence of lymphatic metastases if an appropriate systematic LN dissection is performed, which allows patients to receive adjuvant therapies when nodal metastasis exists (36).

In the study by Zhang *et al.*, two cohorts of patients with early-stage NSCLC, treated by either robotic or VATS anatomical segmentectomy, underwent propensity matching and showed that a significantly higher number of hilar (N1) LNs was harvested in the robotic group (28). Consequently, the robotic system has the potential to improve lymph node dissection, in particular, in peripheral stations, a point that will gain increasing attention if anatomical segmentectomy is demonstrated to be equivalent to lobectomy for stage IA NSCLC. Therefore, dedicated studies on robotic approach for anatomical segmentectomies are required.

This study does have limitations. The trial was closed with a significantly lower number of patients than planned in the design of the study, and no difference between the two arms was demonstrated with regard to the primary outcome (conversion rate and early complications). Additionally, the dropout rate was higher than predicted, probably because in some centers the patients did not undergo preoperative biopsy. Some unavoidable intrinsic characteristics of randomized surgical studies (*i*.*e*., operator skills) and of the surgical techniques (*e*.*g*., number of ports) could induce additional bias in the interpretation of the results.

Despite these aspects, the analysis did show adequate statistical power with regard to secondary outcomes. This result, however, should be considered with caution in the light of the negative result of primary outcome. In the future, we suggest further studies specifically designed to evaluate the performance of minimally invasive techniques for lymph node dissection and the potential improvement of oncological outcome.

In conclusion, we performed the first randomized trial to evaluate the performance of VATS and RATS in the treatment of patients affected by NSCLC. Despite that RATS was not superior to VATS in perioperative outcomes, the robotic technique showed a better performance in LN dissection, which may have potential implications on its oncological efficacy. Further follow-up will be reported in the future regarding long-term outcomes. Larger studies are needed to confirm our results and to compare the role of robotic approach in patients treated with anatomical segmentectomy for early-stage disease.

## Data Availability Statement

The raw data supporting the conclusions of this article will be made available by the authors, without undue reservation.

## Ethics Statement

The studies involving human participants were reviewed and approved by Ethic Committee of Humanitas Clinical and Research Center. The patients/participants provided their written informed consent to participate in this study.

## Author Contributions

GV contributed to conception and design of the study. ED and PM organized the database. GV, AA, EB, AT, CB, SC, LL, MA and PN contributed to data collection. RL and PM performed the statistical analysis. GV and PM wrote the first draft of the manuscript. AA, PN, GP and RL wrote sections of the manuscript. All authors contributed to the article and approved the submitted version.

## Funding

This work was supported by specific grants from the Umberto Veronesi Foundation (Milan, Italy) and Intuitive Surgical Inc. (Sunnyvale, CA, USA). The funders were not involved in the study design, collection, analysis, interpretation of data, the writing of this article or the decision to submit it for publication.

## Conflict of Interest

GV received honoraria from Ab Medica SpA.

The remaining authors declare that the research was conducted in the absence of any commercial or financial relationships that could be construed as a potential conflict of interest.

## Publisher’s Note

All claims expressed in this article are solely those of the authors and do not necessarily represent those of their affiliated organizations, or those of the publisher, the editors and the reviewers. Any product that may be evaluated in this article, or claim that may be made by its manufacturer, is not guaranteed or endorsed by the publisher.
