# Supplementary material for: Perioperative Outcome of Robotic Approach Versus Manual Videothoracoscopic Major Resection in Patients Affected by Early Lung Cancer: Results of a Randomized Multicentric Study (ROMAN Study)
Source: Front Oncol. 2021 Sep 9;11:726408. doi: 10.3389/fonc.2021.726408 (PMC8458770; doi:10.3389/fonc.2021.726408)
Supplement: Supplementary file 1 [file DataSheet_1.docx]

**Supplemental Table S1:** baselines characteristics of the patients enrolled in the VATS and ROBOT groups (Per-protocol analysis).

|  | Group VATS N = 37 | Group ROBOT N = 35 | P value |
| --- | --- | --- | --- |
| Age, years *(mean±SD)* | 68±7.4 | 69±8.7 | 0.80 |
| Female (%) | 15 (41) | 15 (43) | 0.84 |
| BMI *(mean±SD)* | 26±4.2 | 27±4.1 | 0.53 |
| Smoking status | | | |
| *Non smokers (%)* | 10 (42) | 9 (43) | 0.93 |
| *Former (%)* | 13 (48) | 14 (54) | 0.68 |
| *Stop smoking, years median (IQR)* | 15 (5-25) | 20 (6-21) | 0.85 |
| *Smokers (%)* | 14 (58) | 12 (57) | 0.93 |
| *N° cigarettes/day*  *median (IQR)* | 20 (20-30) | 20 (10-30) | 0.28 |
| Pulmonary function evaluation | | | |
| *FEV1, L (mean±SD)* | 91±25.2 | 86±24.9 | 0.36 |
| *DLCO, mMole/min/KPa/L (mean±SD)* | 78±19.4 | 76±20.2 | 0.75 |
| ASA score (%)* |  | |  |
| *I – II* | 24 (65) | 18 (56) | 0.47 |
| *III* | 13 (35) | 14 (44) |  |
| Clinical stage (%)** | | | |
| *IA* | 25 (71) | 27 (77) | 0.48 |
| *IB* | 7 (20) | 6 (17) |  |
| *IIA* | 1 (3) | 2 (6) |  |
| *IIB* | 2 (6) | 0 (0) |  |

Abbreviations: SD, standard deviation; IQR, interquartile range; BMI, body mass index; FEV1, forced expiratory volume in the first second; DLCO, diffusing capacity of the lung for carbon monoxide; ASA score, American Society of Anesthesiology score.

*Data were available for analysis in 69 patients

**Data were available for analysis in 70 patients

**Supplemental Table S2:** Intraoperative characteristics in the VATS and ROBOT groups of patients (Per-protocol analysis).

|  | Group VATS N = 37 | Group ROBOT N = 35 | P value |
| --- | --- | --- | --- |
| Left side (%) | 16 (43) | 14 (39) | 0.71 |
| Lobe (%) | | | |
| *Lower* | 14 (38) | 13 (37) | 0.93 |
| *Middle* | 1 (3) | 6 (17) | 0.055 |
| *Upper* | 22 (59) | 16 (46) | 0.20 |
| Number of incisions, *median (IQR)* | 2 (2-3) | 4 (4-4) | <0.0001 |
| Utility incision size, cm *(mean±SD)* | 3.3±0.67 | 2.7±0.86 | 0.01 |
| Pleural Adhesions (%)* | | | |
| *Light* | 15 (79) | 13 (62) | 0.31 |
| *Moderate* | 4 (21) | 3 (14) | 0.69 |
| *Strong* | 0 (0) | 5 (24) | 0.049 |
| Resection (%) | | | |
| *Lobectomy* | 37 (100) | 34 (97) | 0.49 |
| *Segmentectomy* | 0 (0) | 1 (3) |  |
| R0 (%)** | 35 (95) | 35 (100) | 0.49 |
| R1 (%) | 1 (3) | 0 (0) | 0.99 |
| Operative time (skin to skin), min *(mean±SD)* | 183±40.9 | 179±54.2 | 0.71 |

Abbreviations: SD, standard deviation; IQR, interquartile range.

*Data were available for analysis in 41 patients

**Data were available for analysis in 71 patients. Radicality (R) was assessed following the definition proposed by the IASLC(35,36)

**Supplemental Table S3:** Postoperative outcomes and pathological results in the VATS and ROBOT group of patients (Per-protocol analysis).

|  | Group VATS N = 37 | Group ROBOT N = 35 | P value |
| --- | --- | --- | --- |
| Final Pathology report (%) | | | |
| *Adenocarcinoma* | 31 (84) | 26 (74) | 0.32 |
| *Squamous cell carcinoma* | 3 (8) | 6 (17) | 0.30 |
| *Other* | 3 (8) | 3 (9) | 0.99 |
| Pathological stage (%)* | | | |
| *IA* | 20 (57) | 24 (69) | 0.58 |
| *IB* | 7 (20) | 4 (11) |  |
| *IIA* | 0 (0) | 1 (3) |  |
| *IIB* | 4 (11) | 4 (11) |  |
| *IIIA* | 4 (11) | 2 (6) |  |
| Size, mm *median (IQR)* | 21 (14-30) | 20 (15-28) | 0.54 |
| N° of hilar lymph nodes | | | |
| *Mean±SD* | 4.7±3.6 | 8.3±4.1 | 0.0003 |
| *Median (IQR)* | 4 (2-7) | 7 (6-10) | 0.0001 |
| N° of mediastinal lymph nodes | | | |
| *Mean±SD* | 5.8±3.6 | 8.5±5.4 | 0.005 |
| *Median (IQR)* | 5 (3-7) | 8 (5-12) | 0.0001 |
| N° of Lymph Node Stations Sampled | | | |
| *Mean±SD* | 4.1±1.0 | 5.3±1.4 | 0.0001 |
| *Median (IQR)* | 4 (3-5) | 6 (4-6) | 0.0002 |
| ICU recovery (%) | 4 (11) | 2 (6) | 0.68 |
| ICU stay, days *median (IQR)* | 1 (1-2) | 1 (1-10) | 0.66 |
| Chest tube duration, days *median (IQR)* | 4 (3-6) | 4 (3-7) | 0.63 |
| Hospital stay, days *median (IQR)* | 4 (3-6) | 5 (4-8) | 0.35 |
| Primary outcome** (%) | 10 (27) | 16 (46) | 0.10 |
| Conversion to OPEN (%) | 1 (3) | 3 (9) | 0.36 |
| Early post-operative complications (%) | 9 (24) | 13 (37) | 0.24 |
| Complication grade (%) | | | |
| *I - II* | 4 (13) | 11 (35) | 0.04 |
| *III* | 3 (10) | 2 (9) | 0.99 |
| Most frequent early complication (%) | | | |
| *Air leak* | 4 (11) | 6 (17) | 0.51 |
| *Atrial Fibrillation* | 3 (9) | 4 (11) | 0.71 |
| *Serous drainage* | 1 (3) | 1 (3) | 0.99 |
| *Pneumonia* | 1 (3) | 4 (11) | 0.19 |
| *Pneumothorax* | 1 (3) | 0 (0) | 0.99 |
| *Atelectasis* | 1 (3) | 3 (9) | 0.35 |
| *Urinary tract infection* | 0 (0) | 1 (3) | 0.49 |
| *Other complication* | 2 (5) | 3 (9) | 0.67 |
| Follow- up | | | |
| *Adjuvant Therapy**** | 4 (12) | 3 (10) | 0.75 |
| *Chemotherapy* | 4 (12) | 3 (10) | 0.75 |
| *Radiotherapy* | 2 (6) | 2 (6) | 0.95 |
| *Readmission (%)* | 0 (0) | 4 (16) | 0.13 |
| *Later complication (%)* | 2 (11) | 5 (23) | 0.43 |

Abbreviations: SD, standard deviation; IQR, interquartile range; ICU, intensive care unit.

*Data were available for analysis in 70 patients

**Composite outcome: Conversion to open and/or any early postoperative complication

***Data were available for analysis in 64 patients
